# Psychological well-being and mental health challenges in female university staff

**DOI:** 10.1192/j.eurpsy.2026.11927

**Published:** 2026-07-31

**Authors:** I. Sellami, A. Abbes, A. Feki, M. L. Masmoudi, M. Hajjaji, K. Jmal Hammami

**Affiliations:** 1 occupational medicine; 2 Rheumatoloy, Hedi Chaker Hospital, sfax, Tunisia

## Abstract

**Introduction:**

Female university staff experience notable psychological and mental health challenges, highlighting the need for close mental health monitoring and promotion of workplace well-being.

**Objectives:**

Our study aims to evaluate the mental health among female university staff.

**Methods:**

We conducted a descriptive, analytical and cross-sectional survey to evaluate the mental health of university staff using a self-administered questionnaire. We assessed socio-professional characteristics. Mental health was assessed by the Mental Health Continum Short Form (MHC-SF) questionnaire and the Depression, Anxiety and Stress Scale (DASS-21).

**Results:**

Forty-four female participants were included, with a mean age of 51.8 ± 6.6 years. Most (88.6%) were married, and none were active smokers. The mean job satisfaction score was 7.3 ± 1.9. The mean MHC-SF score was 41.5 ± 16.6, with mean scores for emotional, psychological, and social well-being of 8.4 ± 4.2, 20.1 ± 7.2, and 12.9 ± 6.2, respectively. Anxiety, stress, and depressive symptoms were reported by 52.3%, 38.6%, and 45.4% of participants, respectively. Bivariate analysis showed that overall well-being was negatively correlated with anxiety, depression, and stress (p < 0.05).

**Conclusions:**

Despite moderate job satisfaction, a substantial proportion of female university staff experienced anxiety, stress, and depressive symptoms, which were significantly associated with lower psychological well-being.

**Disclosure of Interest:**

None Declared

